# Acute Effects of Wearing Bite-Aligning Mouthguards on Muscular Strength, Power, Agility and Quickness in a Trained Population: A Systematic Review

**DOI:** 10.3390/ijerph18136933

**Published:** 2021-06-28

**Authors:** Adrià Miró, Bernat Buscà, Joan Aguilera-Castells, Jordi Arboix-Alió

**Affiliations:** Faculty of Psychology, Education Sciences and Sport Blanquerna, Ramon Llull University, 08022 Barcelona, Spain; adriama@blanquerna.url.edu (A.M.); joanac1@blanquerna.url.edu (J.A.-C.); jordiaa1@blanquerna.url.edu (J.A.-A.)

**Keywords:** mouthguards, jaw clenching, vertical dimension, ergogenic effects, neuromuscular performance, sport

## Abstract

The purpose of the present systematic review was to determine the acute effects of wearing bite-aligning mouthguards on muscle strength, power, agility and quickness in athletes. A search of the current literature was performed using the electronic databases (until 1 May 2021) Web of Science, Scopus and Medline. The inclusion criteria were: (1) descriptive design studies; (2) with randomized clinical trials; (3) examining the within-subject acute effects of wearing mouthguards on functional and neuromuscular performance parameters; (4) in physical active, recreational or high-standard athletes. Twenty-seven studies met the inclusion criteria. Sixteen reported positive effects in some of the variables assessed, two reported negative effects and the rest found no significant differences. Overall, the main findings described in the literature are inconclusive concerning the neuromuscular advantages of using mouthguards in muscle strength, power, agility and quickness. These discrepancies might be related to several factors such as differences in testing protocols, poor control of the jaw magnitude and improper mouthguard designs. Despite these differences, after conducting the present systematic review, the authors speculate that jaw clenching while wearing custom-made, bite-aligning oral devices might promote beneficial effects in lower limb power actions, especially in jump ability and knee extension movements. Thus, athletes might consider the use of mouthguards, not only for their protective role but also for the potential ergogenic effects in specific actions, mainly those for which lower limb muscular power are required.

## 1. Introduction

Originally, sports mouthguards were designed to minimize the incidence of orofacial injuries through the absorption of the energy during head and mouth trauma [1]. Besides this preventive role, several studies have investigated the effects of wearing these oral devices on metabolic [2,3,4,5], ventilatory [6,7,8,9], functional [10,11] or neuromuscular performance parameters, and this, concretely, has focused on strength [2,12,13,14,15,16,17,18,19,20,21,22,23,24,25,26,27], power [12,15,16,18,20,21,22,24,25,26,27,28,29,30,31,32,33,34], quickness [26,34,35] or agility [15,25,36]. The potential neuromuscular effects might be attributed to the postural repositioning of the temporomandibular structure and the subsequent muscular rebalancing [37]. This readjustment might promote a more balanced and powered occlusion, thus increasing the effects of the concurrent activation potentiation (CAP) elicited by the remote voluntary contraction (RVC) of the mandible muscles [21,31]. The neuromuscular benefits associated with a RVC might be explained by several mechanisms. One mechanism is based on the integrative function of the cerebral motor cortex and the intercortical connections between the different motor areas of the brain. Thus, when one part of the motor cortex is activated because of jaw clenching, the neural centers of the other parts of the brain are also activated. These centers send impulses to the prime movers which initiates the targeted actions [38]. Another mechanism underlines the increased excitability of spinal motor neurons while an individual clenches the jaw, amplifying the alpha motor neuron activity, gamma loops and muscle spindles, together with descending the cortical input and the stimulus invoked by the afferent input [38]. Furthermore, it is established that jaw clenching increases the excitability of the Hoffman reflex (H-reflex). Indeed, greater force levels in jaw clenching produce greater H-reflex facilitation in some muscle groups, which is evoked with both the descending influence from the cerebral cortex and the afferent input from the oral-facial region [39]. Although several studies [20,21] associated the ergogenic effects of CAP with jaw clenching independent of mouthguard use, others [2,14,24,25] demonstrated beneficial effects when wearing the oral device.

Overall, mouthguards can be classified into three types: standard (or stock), self-adapted (or boil and bite) and customized. The standard type is widely used because of its low cost even though is considered the most uncomfortable and worst adapted to the mouth structure. It is acquired ready to be used, and no fitting process is required. The self-adapted type is a thermoplastic liner that can be fitted to the maxillary teeth after being heated in boiling water to become more malleable. The customized type is the most expensive and requires the expertise of a professional dentist. It is created after an impression or scanning process of the dental structure of the teeth [40]. The use of a certain type or model may directly affect the repositioning of the temporomandibular structure [14] and the comfortability and ability to speak or breathe during exercise [14,29].

To the best of our knowledge, there is no clear evidence of the effects of using mouthguards on neuromuscular performance. While several studies reported benefits due to jaw clenching and the use of mouthguards in power and strength actions, other studies found no beneficial effects, and others even revealed negative effects. Therefore, this systematic review was conducted with the aim to examine the effects of mouthguards on neuromuscular performance in a trained population.

## 2. Materials and Methods

### 2.1. Literature Search

The present systematic review was conducted using the Preferred Reporting Items for Systematic Reviews and Meta-Analysis (PRISMA) statement guidelines according to Moher et al. [41]. Additionally, the study quality of all eligible cross-sectional studies was assessed using the Strengthening Reporting of Observational Studies in Epidemiology (STROBE) criteria described by Vandenbroucke et al. [42]. This analysis consists of a quality scale which combines 22 items of the STROBE checklist and classifies the studies in three categories depending on the obtained score: (1) good quality (> 14 points, low risk of major or minor bias), (2) fair quality (7–14 points, moderate risk of bias) and (3) poor quality (< 7 points, high risk of major bias). Only the good quality studies were included in the review (Table 1). 

To identify the studies for the review, a search on the available literature was performed using the electronic databases Medline, Web of Science and Scopus (until 1 May 2021). The search strategy for each database is described in Table 2. 

### 2.2. Inclusion and Exclusion Criteria

The inclusion criteria admitted (1) studies with a descriptive design, (2) with randomized clinical trials, (3) examining the within-subject acute effects of wearing mouthguards on neuromuscular performance parameters and (4) in physical active, recreational or high-standard athletes. Additionally, studies with untrained/sedentary participants who suffered from some kind of temporomandibular joint (TMJ) disorder or articles with insufficient discussion, poor data presentation and unclear or vague description of the applied protocols were excluded for this systematic review. 

The principal author (A.M.) conducted the data analysis and the search process in major English-language databases without language restrictions. All electronically identified records were evaluated by tittle and abstract. The duplicate articles, which appeared in more than one database, were eliminated and were considered only once. Full texts were obtained for all articles considered to be potentially eligible. Then, the first and second authors (A.M., B.B.) independently examined the preselected records and chose the final studies to be included in the review. In the case of disparity, the third author’s opinion (J.A.-C.) was considered. The studies combining the analysis of neuromuscular parameters with another physiological or perceptive parameter were also included in the review, but only the variables associated with muscular and power performance were considered for the summary of the studies. Likewise, studies which included several types of mouthguards were selected by the present review, but only bite-aligning mouthguard designs were examined. The following information was extracted from each selected study: (1) author and year of publication; (2) sample size, gender and sport status; (3) tests and variables assessed; (4) mouthguards type/conditions and (5) the main findings. 

## 3. Results

### 3.1. Literature Search Results

**Figure 1:**
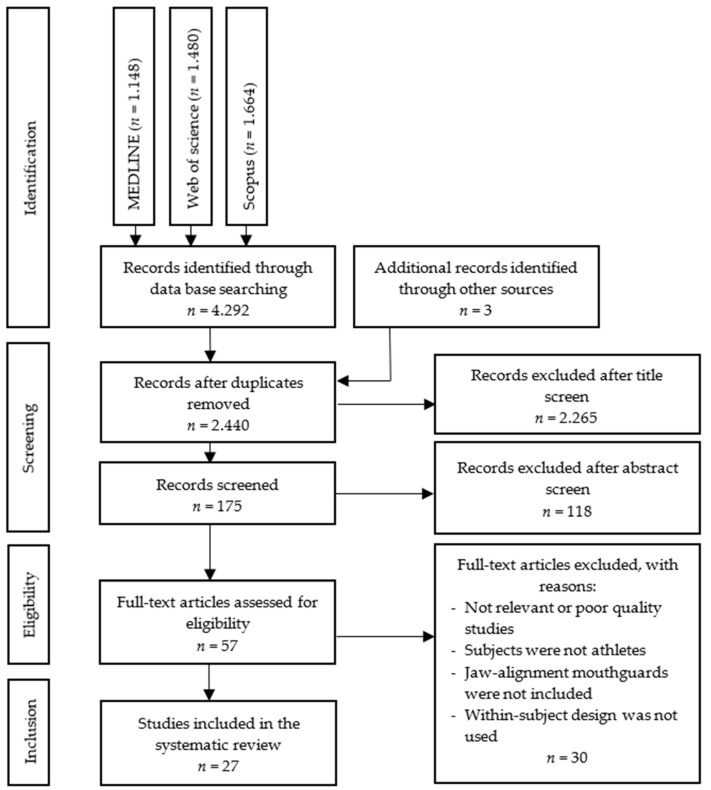
Flowchart of the search and study selection.

A total of 4292 articles were identified after the database screening. All duplicates were excluded using the Mendeley reference manager software (Mendeley Desktop Version 1.19.8, 2008–2020 Mendeley LTd), which left 2440 articles. Additionally, three records were identified through hand research. Then, a manual screening of these articles was made reading the tittle and abstract. After this screening, 2383 were excluded because they did not meet the inclusion criteria. Of these, a total of 57 articles were read entirely and were subjected to the methodological quality analysis. Finally, a total of 27 articles were selected for the final review (Figure 1).

**Figure 2:**
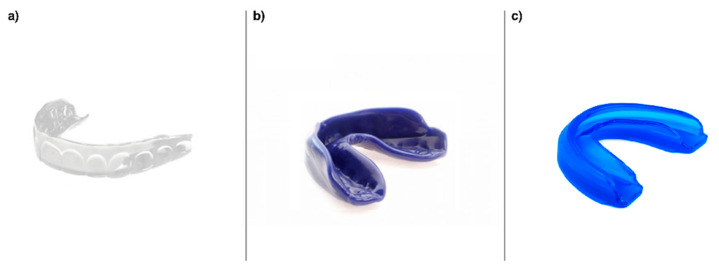
Types of mouthguards described in the systematic review: (**a**) Custom-made MG, (**b**) Self-adapted MG, (**c**) Standard MG.

The summarized data collected from the selected studies are listed in Table 3. The magnitude of differences among conditions is also included in Table 3 but only when the information was reported in the article. All the studies included a within-subject, cross-over design and were specifically developed with athletes who were involved in different sports and standards. Sixteen of these studies [2,12,14,15,16,17,18,20,21,23,24,29,33,34,35,36] were performed with recreational athletes, nine [13,19,22,25,26,27,30,31,43] of them were performed with high-standard athletes and two [28,32] combined both groups. Twenty articles recruited only male participants, [2,12,15,16,17,18,19,20,21,22,23,24,25,27,28,29,30,32,33,43], one recruited only female participants [36] and six recruited participants of both sexes [13,14,26,31,34,35]. The type of mouthguard also varied among studies. The custom-made type (CMM) was the most used in eighteen studies [14,15,16,18,19,22,24,25,26,27,28,29,30,33,34,35,36,43], whereas the self-adapted (SAM) type was used in seventeen [2,12,13,14,15,17,20,21,23,27,28,29,30,31,32,35,36], and the standard (STNDM) in two studies [19,36] (Figure 2). Three of these articles [19,22,43] used placebo (PLA) mouthguards to blind the information of the mouthguard type. Additionally, a comparison between the use of an oral device (MG) with a nonuse condition (NoMG) was reported in ten studies [2,12,13,23,24,25,26,31,32,34], while seventeen [10,14,15,16,17,18,19,20,21,22,27,28,29,30,33,35,43] compared more than one type of mouthguard. Overall, from the twenty-seven reviewed studies, sixteen [2,13,14,17,18,22,23,24,25,26,29,30,31,32,34,35] reported positive effects in some of the variables assessed, whereas two [29,35] reported negative effects. However, the neuromuscular actions were subcategorized to deeply analyze the findings. Thus, most of the studies explored the influence of jaw alignment mouthguards on the dynamic strength and power output, particularly through jump ability, whereas others explored their effect on agility, quickness and isometric and/or isokinetic strength.

### 3.2. Dynamic Strength and Power

The relationship between the use of mouthguards and dynamic strength has been examined in twenty articles [2,12,15,16,18,20,21,22,24,25,26,27,28,29,30,31,32,33,34,35]. On the one hand, upper body muscle strength was evaluated in six of these studies [12,15,22,25,27,35], essentially focusing on bench press and bench throw actions. Two of these articles [25,35] found positive effects. For instance, Dunn-Lewis et al. [35] reported significantly higher bench throw power and force when wearing a CMM compared to the SAM or NoMG condition in recreational team sport athletes. In contrast, the SAM decreased the power output below the NoMG condition. Additionally, Buscà et al. [25] found significantly higher mean power for the CMM rather than NoMG on a 50 kg bench press test in elite basketball players. However, they did not find differences in the 30, 40 or 60 kg loads. All the other studies found no significant differences [12,15,22,27]. 

On the other hand, fifteen out of twenty articles [12,15,18,20,21,22,24,25,26,27,29,31,32,34,35] assessing lower body dynamic strength and power included vertical jumps in their testing protocols, nine of which [18,22,24,25,29,31,32,34,35] found positive effects in at least one of the assessed variables. Eight of these nine studies [18,22,24,25,29,32,34,35] were performed using a CMM. Only one study, led by Duarte-Pereira et al. [29], reported a performance decrease associated with the use of mouthguards. The authors found beneficial effects in the counter movement vertical jump (CMVJ) test when recreational rugby players wore a SAM and CMM compared to the NoMG condition. However, when the athletes wore a CMM, a significant decrease in 15s-RJ height was shown. Lower body power was also evaluated in another nine studies [2,16,22,25,26,28,32,33,35], mainly through knee extension actions and cycle ergometer tests. Cetin et al. [26] investigated the influence of the CMM on WAnT performance with taekwondo athletes, and they found an increased peak and average power while wearing the oral devices. Additionally, Arent et al. [22] compared the effects of two different kinds of CMMs: a neuromuscular dentistry-based mouthguard (nCMM), which involves transcutaneous electric neural stimulation (TENS), and a standard CMM. They found significantly better WAnT performance for the nCMM than the CMM in professional and division I college team sport athletes. In contrast, Jung et al. [16] and Fischer et al. [33] demonstrated no positive effects on this anaerobic test when wearing mouthguards in recreationally trained athletes, despite the last author using an nCMM (with TENS). Moreover, Bourdin et al. [28] also find no significant differences on a 6s-cycling spring test when comparing CMM, SAM, and NoMG in recreational athletes. Moreover, three studies [2,32,35] found positive effects in knee extension actions, whereas one found no significant differences. Dunn-Lewis [35] reported higher power performance in a 3PQ test while wearing a CMM compared to a SAM or NoMG. Additionally, Dudgeon [2] reported better results for the SAM in experienced athletes, who completed more repetitions without assistance on free weight back squats. Additionally, Ebben [32] found a higher power production in a back squat test when recreational athletes clenched their jaw using a SAM compared to a relaxed condition. Nonetheless, Buscà et al. [25] found no differences in a leg-press test with high-standard basketball players wearing a CMM. Finally, on overall dynamic strength test consisting of a three-stroke rowing ergometer, Duddy et al. [30] found a higher maximum power performance when using a CMM compared to a SAM or STNDM.

### 3.3. Isometric Strentgh

Eight studies [17,18,19,20,21,23,24,26] assessed in the review investigated the effects of jaw clenching on isometric strength while wearing a mouthguard. Three studies focused on the upper body [17,23,24], two examined the lower body [20,21] and three other studies [18,19,26] combined upper and lower body tasks. Buscà et al. [24] and Battaglia et al. [23] revealed an increased peak force during a handgrip test in recreationally trained athletes for a SAM compared to NoMG. However, the latest, only found positive effects in the dominant hand, not in the nondominant. Cetin et al. [26] found significant differences in the same test between the CMM and NoMG in elite taekwondo athletes. 

Two studies [19,24] analyzed the influence of wearing mouthguards on the isometric pull arm action test. Buscà et al. [24] showed a higher peak force and RFD during a back row test when recreational athletes wore a CMM compared to NoMG. Nonetheless, Yates et al. [19] found no differences on a two-arm pull test in university football players when compared the PLAM, SAM and STNDM. Moreover, Limonta et al. [17] evaluated the effects of two types of SAM (1 and 3 mm thick) on the maximum isometric strength and fatigue of elbow flexors in physically active volunteers. The authors found a positive effect on isometric force output and neuromuscular efficiency when wearing the occlusal splint. In terms of isometric trunk strength, two articles [18,26] analyzed the influence of mouthguards through a trunk extension test. While Maurer et al. [18] found positive effects due to the CMM in recreational runners, Cetin et al. [26] found no differences in taekwondo elite athletes. 

Four studies [19,20,21,26] did not find significant improvements associated with the use of mouthguards in lower limb isometric strength. Allen et al. [20,21] reported no differences on muscle activity nor force output during a mid-thigh clean pull in recreationally resistance trained males when compared to two types of SAM (a performance SAM and a traditional SAM) with NoMG conditions. Moreover, Yates [19] found no significant differences associated with the CMM on an isometric dead lift with college football players, nor did Cetin [26] on an isometric leg press test with elite taekwondo athletes. Nevertheless, Maurer et al. [18] revealed a higher peak force (both legs) and a better RFD (only right leg) on a leg extension test in recreational runners. 

### 3.4. Isokinetic Strength

Five studies [13,16,19,26,43] investigated the effects of wearing a jaw repositioning mouthguard on isokinetic strength. Two of these studies [19,43] focused on upper limb muscles and three other studies focused on lower limb muscles [13,16,26]. Greenberg et al. [43] found no differences on a shoulder abduction and adduction test when they compared the CMM and PLAM conditions in basketball college athletes. Additionally, Yates et al. [19] also reported no significant differences on an isokinetic upright rowing test in football college athletes.

In terms of lower body strength, Jung et al. [16] showed no significant differences while wearing a CMM on a knee extension and flexion test in college athletes. However, Cetin et al. [26] and Ebben et al. [13] found a higher peak torque for test condition in at least one variable assessed because of wearing mouthguards (CMM and SAM, respectively) in high-standard and college athletes.

### 3.5. Agility/Quickness

The influence of wearing mouthguards on the ability to increase quickness performance was evaluated in three articles [15,25,36], whereas three other articles [26,34,35] focused on agility. Dunn-Lewis et al. [35] and Cetin et al. [26] found no significant differences in 10 and 20 m sprint ability, respectively. Nonetheless, Martins et al. [34] showed a nonsignificant decrease of 4% in 20 m sprint time and a significant decrease of 2% in 40 m when recreational athletes wore a CMM. In this study, the level of significance was set at *p* = 0.01*,* being *p =* 0.04 in the 20 m sprint and *p =* 0.001 in the 40 m. In terms of agility, all the assessed studies demonstrated no significant differences associated with the use of a mouthguard. Buscà et al. [25] in a t-test with professional basketball players, Golem et al. [15] in a Hex test with recreational mixed sport athletes and Queiroz et al. [36] in a shuttle (ball) run test with highly trained footballers all reported no significant improvements when subjects wore mouthguards. Additionally, the last two mentioned studies compared the influence of different types of mouthguards, and they did not find any significant differences.

## 4. Discussion

The main objective of the present systematic review was to analyze the effects of wearing mouthguards on muscular power, strength, agility and quickness. Overall, the main findings described in the literature are inconclusive concerning the neuromuscular advantages of using mouthguards. These discrepancies might be associated with several factors, such as the differences in testing protocols, laboratory equipment, sample characteristics and mouthguard materials or the type used in each study. For instance, several studies used CMMs but others used SAMs or STNDMs. Despite using the same type, the lack of common standards in the manufacturing process produces different mouthguards in terms of design and materials. Therefore, the type of mouthguard used in each study might be a possible explanation for the mentioned discrepancies, as suggested by Bourdin et al. [28]. Furthermore, in some studies [2,12,15,20,21,23], the mouth scanning procedure and the mouthguard manufacturing were conducted by the researchers, while in the rest of the studies they were performed under the expertise of an expert dentist. Another important factor to take into consideration is the subject’s familiarity with oral appliances. Some authors [15,22,35] detailed the previous regular use of mouthguards by the subjects in their studies, whereas others [2,12,17,24,25,33,34,43] indicated that athletes had null or poor experience with the oral devices. However, several studies [13,14,16,18,19,20,21,26,27,28,29,30,31,32,36] did not specify the previous subjects’ experience with mouthguards, even though the practice and level of sport could entail its regular use [19,26,27]. It is possible that mouthguards, being unfamiliar and uncomfortable to most of the nonfamiliarized participants, generated awkward and distracting feelings, affecting the performance and thus leading to the observed differences among the studies. It is suggested that the lack of comfort might make powerful jaw clenching difficult and thus affect the CAP promotion. For this reason, future research should consider long familiarization periods with mouthguards to avoid discomfort and, additionally, to examine the possible long-term adaptations induced by these devices.

The potential effects of mouthguards might be related to the jaw repositioning of the temporomandibular joint and the vertical dimension of occlusion (VDO) [14,23]. The VDO has been defined as the interocclusal distance between dental arches in the maximum intercuspation [44]. It is suggested that an increase in posterior thickness will open the lower airway path and optimize afferent and efferent signaling from the sensorimotor system [14]. The effect of VDO magnitude is unclear and different between individuals [16]. Nonetheless, it is speculated that the distance to achieve the maximum occlusal bite force is about 8 mm between the first molars [37]. Despite the possible enhancing role of an adequate jaw repositioned mouth with the correct vertical dimension on the strength and power output of the prime movers, several studies [2,12,13,15,18,20,21,22,23,25,27,29,31,32,33,34,35,36,43] did not reveal specific manufacturing details such as the splint thickness or the occlusal space elicited by the oral appliances. Indeed, the relevant contribution of the mouthguard’s thickness on the VDO should encourage future researchers to describe these issues. Additionally, the analyzed studies included upper, lower or both dental devices with full or partial coverage, which may also determine the jaw alignment and the distribution of the clenching forces. It has been shown that full coverage, with anterior dental contacts, produces a higher TMJ force because of a longer lever arm [17]. Thus, the different mouthguard designs may influence the mechanical orientation of the jaw, thereby improving physical performance [34,35].

Several authors have demonstrated the relationship between the use of mouthguards with an increased force and muscle activation of the mandible muscles, thus increasing the neuromuscular effects [13,14]. For this reason, future investigations should provide sufficient information about the amount of jaw clenching, both with and without the mouthguard. Indeed, despite not quantifying the forces generated, some authors [20,21,24,25,31,32] encouraged athletes with a specific instruction (i.e., clench as powerfully as possible). However, other authors [12,17,35] did not encourage athletes with any specific instruction about the magnitude of jaw clenching, whereas others [12,15,16,19,22,23,26,27,28,29,30,33,34,36,43] did not mention which instruction was given. Thus, it is difficult to draw a solid conclusion associated with the clenching magnitude while wearing mouthguards. Additionally, while several studies [19,22,43] used placebos or different type of mouthguards [2,12,13,23,24,25,26,31,32], others only compared the use and nonuse of mouthguards. In the latest,, the athletes knew under which condition the test was being performed, and this could affect the subject’s predisposition toward the respective action. Future research could consider a double-blind study design comparing different types of mouthguards with a no-mouthguard condition.

### 4.1. Muscle Power

One of the main findings of the present review was the beneficial effect of jaw clenching while wearing a mouthguard on jump ability. Indeed, 60% of the studies assessing vertical jump reported a meaningfully higher performance when athletes used the intraoral device. Interestingly, all of these studies included the CMM in their testing protocols. Thus, it could be speculated that the lower limb muscle power, measured through vertical jump, might be positively influenced by the use of a CMM. The authors attributed these findings to the potential effect of CAP, which is elicited through the remote voluntary contraction of the mandible muscles [31]. It is believed that the RVC generates a multiphase response characterized by an initial intercortical connection, followed by a supraspinal facilitation, an enhanced H-reflex and a concomitant decrease in the reflex intensity [32,38]. Thus, during power actions, which involve fast stretch-shortening cycles (SSC) and rapid force generation changes, sensory neurons from the muscle spindle send signals through motor neurons to the spine, which communicates with the brainstem. When this information overflows, the stretch reflex is activated, as shown, for instance, during the CMVJ. Muscle spindles are activated during the countermovement because the large muscle groups in the lower limbs are quickly lengthened during the eccentric phase. The muscle spindles communicate to the central nervous system (CNS), which transfers the stimulus to the lower body muscles and promotes a forceful and explosive vertical jump [45]. Nevertheless, Duarte-Pereira et al. [29] reported a performance decrease associated with the use of mouthguards. Although they found beneficial effects on a CMVJ test, the authors reported a significant decrease on 15s-RJ height when recreational rugby players wore a CMM compared to a NoMG condition. This performance decrease might be related to the uncomfortable and restrictive expiratory effects of the mouthguards used, which could negatively impact the airflow path during the 15-RJ test and, thereby, the performance.

Although some authors [20,21] attributed the ergogenic effects of CAP to jaw clenching beyond the use of oral appliances, others [2,24,25] showed enhanced performance when wearing the oral device. In fact, Gage et al. [14] demonstrated that is not possible to produce a maximal jaw contraction with a bare mouth, since depressor muscles are active during clenching to protect the teeth. Moreover, when athletes clench with uncovered teeth, possible imbalances in the temporomandibular musculature could be magnified [10,46]. Thus, clenching bite-aligning intraoral devices seems to produce changes in condylar position and better redistribution of the clenching forces, thus leading to a more highly powered occlusion and further increasing the neuromuscular effects of the jaw clenching [24]. Moreover, this balanced occlusal force distribution, derived by the use of mouthguards, could involve changes in the peripheral proprioceptive input of the orofacial region that may affect the CNS through the trigeminal nerve, after which the CNS transfers the modified output signal via spinal nerves and autonomic nerves to the musculoskeletal system [16].

Other studies assessing lower body dynamic strength focused on cycle-ergometer and knee extension tests. From the five studies including cycle-ergometer tests, two [22,26] found positive effects and three [16,28,33] reported no significant differences when comparing the use and nonuse of mouthguards. It is possible that the mouthguard design used in the latest studies did not produce an optimal VDO to elicit the CAP effects. Fischer et al. [33] did not detail the occlusal space promoted by the mouthguards, but Bourdin et al. [28] and Jung et al. [16] reported a 2 mm increase in VDO when the athletes wore the oral device. This value differs from the 8 mm presented by Arima et al. [37]. In this vein, Bourdin et al. [28] did not find differences between conditions (SAM, CMM and NoMG) on airflow dynamics nor on oxygen uptake (VO_2_), which reinforces the hypothesis that mouthguards did not promote an adequate VDO. From the four studies including knee extension actions, three [2,32,35] reported beneficial effects, and one [25] reported no significant differences. The three studies which found positive effects involved recreational athletes, whereas the study that found no differences involved high-standard athletes. In the last study, professional basketball players performed a leg press test with different loads. The results revealed no significant differences when comparing the use and nonuse of a CMM in any of the leg press loads. The authors attributed these findings to different factors, such as the power test duration (which makes continuous jaw clenching impossible), the different ages of the athletes or the years of experience in weightlifting training. Although the three studies reporting beneficial effects were performed with recreational athletes and the one reporting negative effects was performed with high-standard athletes, it is not possible to conclude that the training standard constitutes a crucial factor for the knee extension actions when athletes used CMMs.

In terms of the upper body dynamic strength, it is difficult to find a relationship between the use of mouthguards and neuromuscular performance. All the studies investigated the effects of wearing mouthguards on the bench press and bench throw actions. Two out of six studies [25,35] reported beneficial effects. However, these two studies showed different results. Concretely, Dunn-Lewis et al. [35] found significantly higher performance in bench throw power and force when wearing a CMM compared to a SAM or NoMG in recreational athletes. Nonetheless, with the SAM, the athletes experienced a significant decrease in power output compared to the NoMG condition. Additionally, Buscà et al. [25] reported higher mean power with the CMM compared to NoMG on a 50 kg bench press test in elite basketball athletes. However, they did not find differences in 30, 40 or 60 kg loads. It is speculated that potential discomfort, reported by some athletes, and the variable response of elite athletes to high neurological activations [47] support this lack of consistency among the final results.

### 4.2. Isometric Strength

From the eight studies examining the isometric muscle strength, four [17,18,23,24] reported positive effects when the athletes used an oral appliance, whereas the other four [19,20,21,26] did not find significant differences. On the one hand, focusing on the lower body strength, only one study [18] found positive effects, and four [19,20,21,26] did not reveal significant differences. Concretely, Cetin et al. [26] found no differences when comparing the use and nonuse of mouthguards on isometric leg and back strength in elite taekwondo athletes. The authors attributed these results to the fact that subjects had never worn a CMM before the study, and no chronic adaptations could be generated. On the other hand, six studies [17,19,23,24,26] examined upper body muscle strength. From these studies, four [17,18,23,24] found beneficial effects due to mouthguard use in recreational or physically active athletes. Concretely, Limonta et al. [17] investigated the effects of two different SAM occlusal splints (1 and 3 mm) with respect to the NoMG condition on the isometric contractions of elbow flexors. Their findings indicated that the use of mouthguards enhanced the maximum isometric strength, lowering force decay and promoting a better neuromuscular efficiency. Additionally, when comparing the 3 mm thick SAM (full coverage) with the 1 mm thick SAM (only posterior coverage), the authors found a lower force decrease in a prolonged maximum contraction for the 3 mm thick SAM condition. The authors maintained that higher mandibular stability may promote a better length and realignment of the occlusion muscles, leading to a higher MVC with a similar EMG activity. Moreover, the authors stated that jaw repositioning can be associated with better postural control, functional proprioception and spinal alignment, promoting higher neuromuscular coordination. Battaglia et al. [23] examined differences between the use and nonuse of mouthguards during an isometric handgrip test in different martial arts athletes. The authors observed a significant increase in peak force while wearing an occlusal splint with the dominant hand, whereas only significant differences were found with the nondominant hand in regular mouthguard users. The authors attributed these findings to better long-term adaptations and to the fact that mouthguards may reinforce more effective regulation on the efferent motor pathways via potentiation of an afferent stimuli from the periodontal mechanoreceptors and muscle spindle fibers activated during teeth clenching with balanced occlusion. In contrast to these findings, two studies conducted with professional athletes [19,26] revealed no significant differences in upper body isometric strength. It is speculated that that the VDO increase promoted by the mouthguard design was less effective in eliciting the CAP effects.

Overall, according to the collected data, it could be hypothesized that the use of mouthguards might improve the upper body isometric strength in recreational athletes or physically active subjects, whereas the studies with high-standard athletes did not reveal significant differences. Nevertheless, no adverse effects were described in any of the reviewed studies.

### 4.3. Isokinetic Muscle Strength

Five studies investigated isokinetic muscle strength, two [19,43] focusing on the upper body and three [13,16,26] on the lower body. One the one hand, the two upper body studies, which were performed with college athletes, found no significant differences. On the other hand, two out of three studies assessing the lower body isometric strength found beneficial effects when wearing a CMM on knee extension actions, whereas one reported no significant differences. The latter [16] did not find significant differences between jaw clenching without a mouthguard and with a full-coverage CMM (2 mm VDO increase) in any of the variables assessed. Indeed, four studies included CMM in their protocol (one also included PLAM), three of which reported no significant differences and one which reported beneficial effects. One study included SAM and also reported beneficial effects. Moreover, four out of five studies involved experienced athletes, with two reporting beneficial effects and two reporting no differences. For this reason, it is difficult to attribute the training standard or the mouthguard type as a relevant factor in determining enhanced isokinetic muscle strength performance. However, an adequate VDO might be considered an important factor in isokinetic strength to be taken into consideration when using mouthguards. Nonetheless, more well-designed studies with accurate information about the mouthguards used and the VDO promoted by these devices are required to search for more solid conclusions in both recreational and high-standard athletes.

### 4.4. Agility/Quickness

Any analyzed study in this systematic review reported beneficial effects on agility because of the use of mouthguards. For instance, Buscà et al. [25] found no significant differences between the use and nonuse of a CMM in the agility t-test in professional basketball players. These findings are in line with Queiroz et al. [36], who tested a shuttle-run with ball test in female soccer players, and Golem and Arent [15], who also found no significant differences between conditions in a HEX agility test in collegiate male athletes. The authors attributed these results to the complexity of the neuromuscular processes and the coordinative demands during agility tests. Moreover, the duration and the nature of these kinds of tests makes the continuous RVC of the mandible muscles and the consequent potential effects of the CAP impossible.

In terms of quickness, two out of three studies found no significant differences in a 10 and 20 m sprint test, while one reported positive effects in a 40 m test. In this vein, Martins et al. [34] sustained a potential correlation between vertical and horizontal power production and maximal speed. In fact, the authors also found a nonsignificant 3% increase in vertical power and a significant 2% in horizontal power, thus explaining the decrease in 40 m sprint. This is in line with the nonsignificant differences reported by Cetin et al. [26] on a 20 m sprint test, which also showed no benefits in horizontal and vertical lower limb power.

All tests included the CMM in their testing protocol, so it is difficult to draw a solid conclusion related to mouthguard use in agility or sprint actions. Moreover, three tests were performed with high-standard athletes and three with recreational athletes. Thus, it is also difficult to link the training standard to any potential ergogenic effect on agility or sprint ability.

## 5. Conclusions

After conducting a detailed systematic review, the authors conclude that the acute effects of jaw clenching while wearing mouthguards on muscle strength, power, agility and quickness are inconclusive. It is shown that the use of mouthguards might promote beneficial effects in lower limb muscular power, especially in jump ability and knee extension actions. These findings are not extensive to agility, quickness or isometric or isokinetic muscular actions, the studies of which did not report consistent results about the potential benefits of wearing mouthguards on athletic performance. This lack of conclusiveness might be related to several factors, such as differences among the testing protocols, poor control or quantification of the jaw magnitude and different mouthguard designs.

The present systematic review might also conclude that custom-made mouthguards showed better results than self-adapted or standard types and, overall, do not negatively affect athletic performance. Thus, in addition to their protective role, clinicians and practitioners can consider the use of dentistry-designed mouthguards in sports, mainly in those for which lower limb muscular power is required.

## Figures and Tables

**Table 1:** The study quality analysis (STROBE checklist).

Reference	Title and Abstract	Introduction		Methods									Results					Discussion				Other Information	Strobe Points
	1	2	3	4	5	6	7	8	9	10	11	12	13	14	15	16	17	18	19	20	21	22	
Dudgeon et al. 2017 [2]	+	+	+	+	+	+	+	+	-	-	+	+	+	+	+	+	+	+	-	+	+	+	19
Allen et al. 2014 [12]	+	+	+	+	-	+	+	+	-	-	+	+	-	-	+	+	+	+	+	+	+	-	16
Ebben et al. 2010 [13]	+	+	+	+	-	-	-	+	-	+	+	-	+	+	+	+	+	+	+	+	-	+	16
Gage et al. 2015 [14]	+	+	+	+	+	+	-	+	-	-	+	+	-	+	+	+	+	+	-	+	+	+	17
Golem et al. 2015 [15]	+	+	+	+	+	+	+	+	-	-	+	+	+	+	+	+	-	+	+	+	+	+	19
Jung et al. 2013 [16]	+	+	+	+	+	+	+	+	-	-	+	+	-	-	+	+	-	+	-	+	+	-	15
Limonta et al. 2017 [17]	+	+	+	+	+	+	-	+	+	+	+	+	+	+	+	+	+	+	+	+	+	+	21
Maurer et al. 2018 [18]	+	+	+	+	+	+	-	+	+	+	+	+	+	+	+	+	+	+	+	+	+	+	21
Yates et al. 1984 [19]	-	+	+	+	+	+	-	+	-	-	-	+	+	-	+	+	+	+	-	+	+	+	15
Allen et al. 2016 [20]	+	+	+	+	-	+	+	+	-	+	+	+	-	-	+	+	+	+	-	+	+	-	16
Allen et al. 2018 [21]	+	+	+	+	+	+	+	+	-	-	+	+	-	-	+	+	-	+	+	+	+	+	17
Arent et al. 2010 [22]	+	+	+	+	+	+	+	+	-	-	+	+	-	-	+	+	+	+	-	+	+	+	17
Battaglia et al. 2018 [23]	+	+	+	+	-	+	+	+	-	-	+	+	-	-	+	+	-	+	+	+	+	+	16
Buscà et al. 2016 [24]	+	+	+	+	+	+	+	+	-	-	+	+	+	-	+	+	-	+	+	+	+	+	18
Buscà et al. 2018 [25]	+	+	+	+	+	+	+	+	-	-	+	+	-	-	+	+	-	+	+	+	+	+	17
Cetin et al. 2009 [26]	+	+	-	+	+	-	+	+	-	-	+	-	+	+	+	+	-	+	+	+	+	+	16
Drum et al. 2016 [27]	+	+	+	+	+	-	+	+	-	-	+	+	+	-	+	-	+	+	+	+	+	+	17
Bourdin et al. 2006 [28]	+	+	-	+	+	-	+	+	-	-	+	+	+	-	+	+	-	+	-	+	+	+	15
Duarte-Pereira et al. 2008 [29]	+	+	+	+	+	+	+	+	-	-	+	+	-	-	+	+	+	+	-	+	-	-	15
Duddy et al. 2012 [30]	+	+	+	+	-	+	+	+	+	+	+	+	+	+	+	+	+	+	-	-	+	-	18
Ebben et al. 2008 [31]	+	+	+	+	-	-	-	+	-	+	+	-	+	+	+	+	+	+	+	+	-	+	16
Ebben et al. 2010 [32]	+	+	+	+	-	-	-	+	-	+	+	-	+	+	+	+	-	+	+	+	-	+	16
Fisher et al. 2017 [33]	+	-	+	+	-	-	+	-	-	-	+	+	+	+	+	+	+	+	+	+	+	-	15
Martins et al. 2018 [34]	+	+	+	+	+	+	+	+	+	-	+	+	+	+	+	+	+	+	+	+	+	+	21
Dunn-Lewis et al. 2012 [35]	+	+	+	+	+	+	+	+	+	-	+	+	+	-	+	+	+	+	-	+	+	+	19
Queiroz et al. 2013 [36]	+	+	+	-	-	+	+	+	-	-	+	+	-	-	+	+	+	+	+	+	+	-	15
Greenberg et al. 1981 [43]	-	+	+	+	+	+	-	+	-	-	-	+	+	-	+	+	+	+	-	+	+	+	15

**Table 2:** Search strategy and databases used.

Database	Search Strategy
-Web of Science (*n* = 1.480)	#TOPIC 1: mouthguard* or “mouth Guard*” or mouthpiece* or “mouth piece*” or “oral appliance*” or “oral splint*” or “bite splint*” or “intraoral device*” or “intraoral appliance*” or “intraoral splint*” or “over-the-counter jaw-repositioning*” or “jaw repositioning” AND #TOPIC 2: sport* or athlete* or exercise or strength or force or muscular or muscle or power or anaerobic or neuromuscular or activation or agility or jump
Scopus (*n* = 1.664)	TITLE-ABS-KEY (mouthguard* OR mouthpiece* OR *“oral device”* OR “*intra-oral device”* OR *“oral appliance”* OR *“intraoral appliance”* OR *“jaw repositioning”*) AND TITLE-ABS-KEY (sport* OR athlete* OR exercise OR strength OR *Force OR muscular* OR *muscle* OR *power* OR an*aerobic* OR *neuromuscular OR activation* OR *agility* OR *jump*)
MEDLIN-PubMed - (*n* = 1.148)	[Title/Abstract] (mouthguard* OR “mouth Guard*” OR “mouth piece*” OR mouthpiece* OR “oral appliance*” OR “oral splint*” OR “oral splints” OR “bite splint*” OR “intraoral device*” OR “intraoral appliance*” OR “intraoral splint*” OR “jaw repositioning” OR “interocclusal device) AND [Title/Abstract] (sport* OR athlete* OR exercise OR neuromuscular OR strength OR force OR muscular OR muscle OR power OR activation OR agility OR jump)

**Table 3:** Summary of the collected data on acute effects of mouthguards in muscle strength, power, agility and quickness actions.

Author/Year	Sample	Type of Test	Dependent Variables	Type of MG/Condition	Findings
Dudgeon et al. 2017 [2]	15 (men) Experienced athletes	6 × 10 reps back squats at 80% of 1 RM	Weight lifted (kg) and No. reps	Cond. 1: SAM Cond. 2: NoMG	SAM (vs NoMG): ↑* repetitions completed without assistance and ↓* assisted repetitions.
Allen et al. 2014 [12]	21 (men) Recreational athletes; no sport specification	CMVJ and 1RM BP	CMVJ height (inch), RFD (N/ms) and PF (N)/BP 1RM (lbs)	Cond. 1: SAM Cond. 2: NoMG	No #* in any variable of CMVJ or BP. No ↓* performance.
Ebben et al. 2010 [13]	23 (men and women) College athletes; team sports	Isokinetic knee flex-ext. (EMG)	PT, RTD, P, work and %EMG	Cond. 1: SAM Cond. 2: NoMG	SAM (vs NoMG): overall ↑* PT ($\eta_{p}^{2}$ = 0.51) and P in knee ext. ($\eta_{p}^{2}$ = 0.5), ↑* prime mover’s %EMG ($\eta_{p}^{2}$ = 0.25); only men ↑* RTD ($\eta_{p}^{2}$ = 0.28) and work in knee ext. ($\eta_{p}^{2}$ = 0.34), ↑* PT ($\eta_{p}^{2}$ = 0.23) and P ($\eta_{p}^{2}$ = 0.23) in knee flex.; only women ↓* flexor digitorum %EMG in all tests.
Gage et al. 2015 [14]	24 (men and women) Recreational athletes; weightlifters	75% Power Clean Lift (EMG)	Interocclusal distance (mm), ATM, MAS, cervical paraspinal and SCM mean and peak %MVIC (mV)	Cond. 1: SAM [5.3 mm] Cond. 2: SAM [3.5 mm] Cond. 3: CMM [3.69 mm] Cond. 4: NoMG [3.54 mm]	SAM [5.33 mm] (vs SAM [3.5 mm]/CM/NoMG): ↑* occlusal distance MG (vs NoMG): ↑* mean %MVIC MAS, ATM and SCM; ↑*peak%MAS SAM [5.33] (vs CMM/NoMG): ↑* mean %MVIC ATM and SCM SAM [5.33] (vs SAM [3.5 mm]/NoMG): ↑* mean and peak %MVIC MAS CMM (vs SAM [3.5 mm]): ↑* mean %MVIC ATM and MAS
Golem et al. 2015 [15]	22 (men) Recreational athletes; martial and team sports	VJ, 3 RM BP, HEX agility test	VJ height (cm) and power (w)/3RM BP (Kg)/HEX agility test (S)	Cond. 1: SAM Cond. 2: CMM Cond. 3: PLAM Cond. 4: NoMG	PLAM vs. SAM vs. CMM vs. NoMG: No #* in VJ height and P, agility or BP strength
Jung et al. 2013 [16]	20 (men) Recreational athletes; no sport specification	Isokinetic knee flex-ext. and WAnT	Isokinetic strength (Nm), muscular P (w) and muscular endurance (joules)/WAnT PP (w) and rate to fatigue (w/s)	Cond. 1: CMM [2 mm] Cond. 2: NoMG	CMM (vs NoMG): No #* in WAnT, max. isokinetic strength, P and muscular endurance of knee joint during flex-ext. movements.
Limonta et al., 2017 [17]	9 (men) Recreational athletes; no sport specification	Elbow flexors MVIC/60 s MVIC at 100%/80% MVIC until exhaustion	MVIC (N), NE (N/mV), EMG RMS (mV) and EMG mean Frequency (meanF) (Hz)/80%exhau t of F in target (t-target) (s), F distance (%) from target (ΔF) and F CoV (%)/100%60 s force decay (%Δfi-Fe)	Cond. 1: SAM Post [1 mm] Cond. 2: SAM [3 mm] Cond. 3: NoMG	SAM [3 mm]/SAM [1 mm] (vs NoMG): ↑* F and NE, ↓* EMG MF in MVC; No #* in 80%exh whereas ↑ t-target in 80%exh SAM [3 mm] (vs SAM [1 mm]/NoMG): ↓* of (Δfi-Fe) in 100%60 s
Maurer et al. 2018 [18]	23 (men) Recreational athletes; runners	SJ, CMVJ, DJ (32 and 40 cm), Isometric trunk flex-ext. and isometric LP	VJ height (cm) and contact time (only DJ)/RFD (N/s)/Isometric max. F (N)	Cond. 1: relax CMM in centric occlusion (CMMc) Cond. 2: CMM myocentric position (CMMd) Cond. 3: max. inter-cuspidation (CMMm) Cond. 4: occlusion at rest (NoMG)	CMMc/CMMd (vs NoMG/CMMm): ↑* Squat, CMVJ, DJ32 and DJ40. ↑* trunk ext., leg press force and RFD.No #* in symmetry between flex-ext. ↑ condyle central position and ↑ strength and speed-strength parameters.
Yates et al. 1984 [19]	14 (men) College athletes; football	isokinetic up-right row, Isometric dead lift and arm pull,	Force (N)	Cond. 1: CMM [2–3 mm] Cond. 2: PLAM Cond. 3: STNDM	CMM vs. PLAM vs. STNDM: No #* in any variable assessed
Allen et al. 2016 [20]	36 (men) Recreational athletes; no sport specification	CMVJ and IMTCP	Peak EMG signal (mV) G, H, VMO	Cond. 1: SAMp + jaw Cond. 2: SAMp + relax Cond. 3: SAMt + jaw Cond. 4: SAMt + relax Cond. 5: NoMG + jaw Cond. 6: NoMG + relax	MG (vs NoMG): No #* % EMG in CMVJ or MTCP. SAMp/NoMG (vs SAMt) ↑* % EMG (G, H and VMO) in CMVJ.Jaw (vs Nonjaw) ↑* % EMG (G, H and VMO) in CMVJ. No #* in MTCP.
Allen et al. 2018 [21]	36 (men)Recreational athletes; no sport specification	CMVJ and IMTCP	PF and normalized PF (nPF) (N), RFD (N/s) and Jump height (cm)	Cond. 1: SAMp + jaw Cond. 2: SAMp + relax Cond. 3: SAMt + jaw Cond. 4: SAMt + relax Cond. 5: NoMG + jaw Cond. 6: NoMG + relax	SAMp vs. SAMt vs. NoMG: No #* in any variable of CMVJ or MTCP. Jaw (vs Nonjaw): ↑* in PF ($\eta_{p}^{2}$ = 0.31), nPF ($\eta_{p}^{2}$ = 0.27) and RFD ($\eta_{p}^{2}$ = 0.27) during IMTCP.
Arent et al. 2010 [22]	22 (men)Professional and college athletes; team sport and martial arts	VJ, BP and WAnT + 8 x10 s interval	VJ height (cm)/BP (reps.)/WAnT PP and mean(W/kg)	Cond. 1: neuromuscular CMM (with TENS) Cond. 2: CMM	nCMM (vs CMM): ↑* in VJ (ES = 0.27), 30 s WAnT PP (ES = 0.33), WAnT + intervals PP (ES = 0.42) and meanP. (ES = 0.3) No #* in BP (ES = 0.05) or 30 s WAnT meanP (ES = 0.1).
Battaglia et al. 2018 [23]	25 (men) Recreational athletes; martial arts	Handgrip	Handgrip force (Kg)	Cond. 1: SAM Cond. 2: NoMG	SAM (vs NoMG): ↑* in dominant hand PF. No #* in non-dominant hand PF.
Buscà et al. 2016 [24]	28 (men) Recreational athletes; team sports	Handgrip, BRW and CMVJ	Handgrip PF (N)/BRW-PF (N) and RFD (N/s)/CMVJ height (cm) and meanP (N)	Cond. 1: CMM [5.4 mm] Cond. 2: JAW Cond. 3: Non-JAW	CMM (vs JAW/Non-JAW): ↑* in HG-PF ($\eta_{p}^{2}$ =0.584), BRW-PF ($\eta_{p}^{2}$ = 0.337), BRW-150 ($\eta_{p}^{2}$ = 0.332), BRW-300 ($\eta_{p}^{2}$ = 0.251), BRW-450 ($\eta_{p}^{2}$ = 0.308), CMVJ meanP ($\eta_{p}^{2}$ = 0.23) and CMVJ height ($\eta_{p}^{2}$ = 0.285).JAW (vs Non-JAW): ↑* in HG-PF.
Buscà et al. 2018 [25]	13 (men) High-standard athletes (basketball players)	CMVJ, CMVJa, T-Test, BP and LP	CMVJ and CMVJa height (cm) and P (W)/T-Test (s)/BP and LP PV (m/s), TTPP (ms), avg. P (W), V (Km/h) and F (N).	Cond. 1: CMM Cond. 2: NoMG	CMM (vs NoMG): ↑* in CMVJ height (ES = 0.21), CMVJ P (ES = 0.21), CMVJa height (ES = 0.26) and 50 kg BP (ES = 0.24). CMM (vs NoMG): No #* in CMVJa P (ES = 0.12), agility T-test (ES = -0.44), none of the leg press loads nor of the rest BP loads.
Cetin et al. 2009 [26]	21 (men and women) High-standard athletes; taekwondo	SJ, CMVJ, WAnT, Isokinetic leg, isometric back-leg, handgrip and 20 m-sprint.	SJ and CMJ height (cm)/WAnT PP and avg. P (w/Kg)/Isokinetic PT (Nm)/Isometric back and leg strength (kg)/handgrip (kg)/20 m-sprint (s)	Cond. 1: CMM Cond. 2: NoMG	CMM (vs NoMG): No #* in SJ, CMVJ, isometric leg and back strength, handgrip or 20 m-sprint time. CMM (vs NoMG): ↑* in WAnT PP and avg. P and ↑* concentric hamstring PT.
Drum et al. 2016 [27]	10 (men) College athletes; football players	CMVJ, SJ and 1RM BP	CMVJ and SJ (cm)/1RM BP (lbs)	Cond. 1: SAM Cond. 2: CMM Cond. 3: NoMG	CMM vs. SAM vs. NoMG: No #* in SJ ($\eta_{p}^{2}$ = 0.12), CMVJ ($\eta_{p}^{2}$ = 0.15) or 1RM ($\eta_{p}^{2}$ = 0.1).
Bourdin et al. 2006 [28]	19 (men) High-standard and recreational athletes; team sports	6 s cycle ergometer sprints	F (N), V (m/s) and P (W)	Cond. 1: SAM Cond. 2: CMM [2–2.5 mm] Cond. 3: NoMG	SAM vs. CMM vs. NoMG: No #* in F, V and P output.
Duarte-Pereira et al., 2008 [29]	11 (men) Recreational athletes; rugby players	CMVJ and 15s-RJ	CMVJ and RJ height (cm)/RJ No. reps and avg. P (W)	Cond. 1: SAM Cond. 2: CMM [4 mm] Cond. 3: NoMG	CMM (vs NoMG): ↑* CMVJ ↓* RJ 15 s height.SAM vs. CMM: No #* in CMVJ.SAM/CMM (vs NoMG): No #* in RJ 15s P.
Duddy et al. 2012 [30]	18 (men) High-standard athlete; rowers	3-stroke ergometer	3-stroke max. P (W)	Cond. 1: SAM Cond. 2: CMMCond. 3: NoMG	CMM (vs SAM): ↑* Power in the 3-stroke test. CMM/SAM (vs NoMG): No #* Power in the 3-stroke.
Ebben et al. 2008 [31]	14 (men and women) College athletes; track and team sports	CMVJ	RFD (N/s), TTPF (ms) and PF (N)	Cond. 1: SAM Cond. 2: NoMG	SAM (vs NoMG): ↑* RFD and ↓* TTPF.No #* in PF but suggested beneficial effect.
Ebben et al. 2010 [32]	13 (men)Recreational and college athletes; track and team sports	Back Squat and SJ	Back Squat GRF (N), RFD(N/S) and SJ height (m), GRF (N), RFD (N/s)	Cond. 1: SAM Cond. 2: NoMG	SAM (vs NoMG) = ↑* Back squat GRF ($\eta_{p}^{2}$ = 0.45) and RFD-100 ($\eta_{p}^{2}$ = 0.18), ↑* SJ GRF ($\eta_{p}^{2}$ = 0.27) and RFD-100 ($\eta_{p}^{2}$ = 0.32); ↑* SJ peak RFD ($\eta_{p}^{2}$ = 0.51) and height ($\eta_{p}^{2}$ = 0.34).
Fisher et al. 2017 [33]	23 (men) Recreational athletes; different sports	WAnT	PP (W), Min. P (W), P drop (W), Avg. P (W) and TTPP (s)	Cond. 1: Neuromuscular CMM (with TENS) Cond. 2: CMMCond. 3: NoMG	nCMM vs. CMM vs. NoMG: No #* in TTPP ($\eta_{p}^{2}$ = 0.008), PP ($\eta_{p}^{2}$ = 0.009), min. P ($\eta_{p}^{2}$ = 0.056), P drop ($\eta_{p}^{2}$ = 0.011) and Avg. P ($\eta_{p}^{2}$ = 0.068).
Martins et al. 2018 [34]	24 (men and women) Recreational athletes; no sport specification	CMVJ, CBJ, 20 and 40 m sprint time	CMVJ height (cm) and vertical Power (W)/CBJ distance (m) and horizontal P (W)/20 and 40 m time (s)	Cond. 1: CMM Cond. 2: NoMG	CMM (vs NoMG): ↑ in Vertical P (ES = 0.1), ↑* in Horizontal P (ES = 0.1), ↑* in 40 m sprint (ES = 0.21) and ↑ 4% in 20 m sprint (ES = 0.6).
Dunn-Lewis et al. 2012 [35]	50 (men and women) Recreational athletes; team sports	CMVJ, 10-m sprint, bench throw and 3PQ	CMVJ height (cm), RFD (W/s) and PP (W)/10-m sprint (s)/Bench Throw P (W)/3PQ F (N)	Cond. 1: SAM Cond. 2: CMM Cond. 3: NoMG	CMM vs. SAM vs. NoMG: No #* 10-m sprint time. CMM (vs SAM/NoMG): ↑* in Bench throw P and F (men and women); Only men ↑* in 3PQ P and F; VJ_RFD; No #* in PP or VJ height, despite ↑ in magnitude. SAM (vs NoMG): ↓* bench throw P in men.
Queiroz et al. 2013 [36]	25 (women) Recreational athletes; soccer	Shuttle Run test with ball	Time to finish the test (s)	Cond. 1: SAM Cond. 2: CMM Cond. 3: STNDM Cond. 4: NoMG	SAM vs. CMM vs. STNDM vs. NoMG: No #* in shuttle run test with ball.
Greenberg et al. 1981 [43]	14 (men) College athletes; basketball	Isokinetic shoulder abd-add	Peak torque performance (ft.-lbs)	Cond. 1: CMM Cond. 2: PLAM Cond. 3: NoMG	CMM vs. PLAM vs. NoMG: No #* in any variable assessed

*: significance; #: difference; ↑: increase; ↓: decrease; $\eta_{p}^{2}:\;$ partial eta squared; 3PQ: plyo press power quotient; ATM: anterior temporalis; BP: bench press; BRW: back-row; CBJ: countermovement broad jump; CMM: custom-made mouthguard; CMVJ: countermovement jump; CMVJa: countermovement jump with arms; DJ: drop jump; EMG: electromyography; ES: effect size; F: force; Ft: foot; G: gastrocnemius; GRF: ground reaction force; H: hamstring; HEX: hexagon agility test; IMTCP: isometric mid-thigh clean pull; JAW: jaw clenching; lbs: pounds; LP: leg press; MAS: masseter; MG: mouthguard; MVC: maximum voluntary contraction; MVIC: maximal voluntary isometric contraction; N: Newton; No. reps: number of repetitions; NE: neuromuscular efficiency; P: power; PLAM: placebo mouthguard; PT: peak torque; RFD: rate of force development; RJ: rebound jump; RM: maximum repetition; RMS: root mean square; RTD: rate of torque development; s: seconds; SAM: self-adapted mouthguard; SAMp: self-adapted performance mouthguard; SAMt: self-adapted traditional performance; SCM: sternocleidomastoid; SJ: squat jump; STNDM: standard mouthguard; TTPF: time to peak force; TTPP: time to peak power; V: velocity; VJ: vertical jump; VMO: vastus medialis; W: watts; WAnT: Wingate anaerobic test.
